# Development of a two-circular RNA panel as potential prognostic biomarker for gastric cancer

**DOI:** 10.1186/s12967-021-03075-y

**Published:** 2021-10-02

**Authors:** Jing Liu, Xingwu Zhu, Meinan Yan, Hui Li

**Affiliations:** 1 Department of Gastrointestinal Cancer Biology, Tianjin Medical University Cancer Institute and Hospital, Tianjin, China; 2 Key Laboratory of Cancer Immunology and Biotherapy, Tianjin, China; 3 National Clinical Research Center for Cancer, Tianjin, China

**Keywords:** Gastric cancer, Circular RNA, Biomarker, Prognostic assessment, circPanel

## Abstract

**Background:**

Circular RNAs (circRNAs) have attracted increasing attention in recent years for their potential application as disease biomarkers due to their high abundance and stability. In this study, we attempted to screen circRNAs that can be used to predict postoperative recurrence and survival in patients with gastric cancer (GC).

**Methods:**

High-throughput RNA sequencing was used to identify differentially expressed circRNAs in GC patients with different prognoses. The expression level of circRNAs in the training set (n = 136) and validation set (n = 167) was detected by quantitative real-time PCR (qRT-PCR). Kaplan–Meier estimator, receiver operating characteristic (ROC) curve and cox regression analysis were used to evaluate the prognostic value of circRNAs on recurrence-free survival (RFS) and overall survival (OS) in GC patients. CeRNA network prediction, gene ontology (GO) and Kyoto Encyclopedia of Genes and Genomes (KEGG) analyses were performed for the circRNAs with prognostic significance.

**Results:**

A total of 259 differentially expressed circRNAs were identified in GC patients with different RFS. We found two circRNAs (hsa_circ_0005092 and hsa_circ_0002647) that highly expressed in GC patients with good prognoses, and subsequently established a predictive model for postoperative recurrence and prognosis evaluation, named circPanel. Patients with circPanel^low^ might have shorter recurrence-free survival (RFS) and overall survival (OS). We also performed circRNA-miRNA-mRNA network prediction and functional analysis for hsa_circ_0005092 and hsa_circ_0002647.

**Conclusions:**

CircPanel has the potential to be a prognostic biomarker in GC patients with greater accuracy than a single circRNA and certain traditional tumor markers (e.g., CEA, CA19-9 and CA724).

**Supplementary Information:**

The online version contains supplementary material available at 10.1186/s12967-021-03075-y.

## Background

Gastric cancer (GC) is the fifth most common cancer and the third leading cause of cancer deaths worldwide [1]. Due to GC’s high heterogeneity and the absence of specific symptoms, GC patients are often found at an advanced stage. Surgical resection, combined with adjuvant radiotherapy and chemotherapy, remains the major treatment strategy for GC. Nevertheless, advanced GC patients are still at high risk for postoperative recurrence, often accompanied by rapid disease progression and a poor survival rate [2]. Therefore, it is of great significance to evaluate the postoperative recurrence risk of GC patients in advance of treatment decisions. The current assessment of GC prognostic is mainly based on pathological classification and TNM staging [3]. Some traditional tumor biomarkers, including carcinoembryonic antigen (CEA), carbohydrate antigen 19-9 (CA19-9) and carbohydrate antigen 724 (CA724), only showed limited accuracy in prognostic evaluations [4, 5]. Therefore, developing novel and effective biomarkers for the prognostic assessment of GC patients has always been the goal of the majority of researchers.

Circular RNA (circRNA) is a new type of RNA molecule with a covalently closed structure. Since their discovery in 1976 [6], the formation mechanism, expression characteristics and biological functions of circRNAs have been gradually revealed. Unlike linear mRNAs, circRNAs are mostly derived from back-splicing of precursor mRNAs, and they are widely expressed in mammals with conserved sequences [7]. The circular structure of circRNAs causes them to lack 5′ and 3′ polarity, preventing them from being degraded by exonuclease and resulting in a longer half-life. Moreover, the expression of circRNAs is generally tissue-, cell- and developmental stage-specific. Therefore, circRNAs have been considered as potential disease biomarkers [8–10]. In addition, circRNAs have been found to regulate gene expression at the transcriptional, posttranscriptional and translational levels through a variety of pathways. They can competitively bind microRNAs (miRNAs) to further inhibit their regulation of downstream target genes as ceRNA (competing endogenous RNA), and can be used as protein scaffolds to participate in the activation or segregation of proteins [11]. A few circRNAs also have the potential to be translated into polypeptides or proteins [12]. Abnormal expression of circRNAs has been found in a variety of cancers [13], including lung cancer, liver cancer, breast cancer, colorectal cancer and gastric cancer.

Considering the correlation between circRNAs and gastric cancer, we speculated that circRNAs could serve as biomarkers for prognostic evaluation of GC. In this study, we revealed the circRNA expression profiles of GC patients with different prognoses and established a postoperative recurrence model (circPanel), based on hsa_circ_0005092 and hsa_circ_0002647.

## Methods

### Patients and samples

A total of 313 samples from GC patients were collected from January 1, 2010, to December 31, 2015, at Tianjin Medical University Cancer Institute and Hospital for discovery (n = 10), training (n = 136) and validation (n = 167). All of the patients met the following criteria: (1) pathologically diagnosed with gastric adenocarcinoma; (2) without preoperative radiotherapy or chemotherapy; (3) without a history of other cancers; and (4) without severe postoperative complications (including infection, bleeding and anastomotic fistula). Tumors were staged according to the 8th edition of the American Joint Committee on Cancer tumor-node-metastasis (TNM) staging system. The detailed clinicopathological characteristics of the training and validation sets are summarized in Additional file 4: Table S1.

### RNA sequencing and bioinformatics analysis

Tumor tissue-derived RNAs from 10 GC patients with extreme prognosis (5 patients with RFS ≤ 1 year and 5 patients with RFS > 3 years) were used for circRNA sequencing. The clinicopathological information of the two groups was matched. A total amount of 5 μg RNA per sample was used as input material for the RNA sample preparations. Firstly, ribosomal RNA was removed by Epicentre Ribozero™ rRNA Removal Kit (Epicentre, USA), and rRNA free residue was cleaned up by ethanol precipitation. Subsequently, the linear RNA was digested with 3 U of RNase R (Epicentre, USA) per μg of RNA. The sequencing libraries were generated by NEBNext® Ultra™ Directional RNA Library Prep Kit for Illumina® (NEB, USA) following manufacturer’s recommendations. At last, products were purified (AMPure XP system) and library quality was assessed on the Agilent Bioanalyzer 2100 system. The original image data obtained from sequencing were translated into sequenced reads through base calling analysis. The sequencing error rate of each sample was less than 0.1% (Additional file 3: Figure S3). circRNAs were identified by the find_circ_ tool [14]. The circRNAs currently known were named with circBase ID, while novel circRNAs were annotated as hg19_circ_xxxxxxx with chromosome position and splicing sequences. The expression of circRNAs in each sample was counted and normalized by the transcripts per million (TPM). Volcano plots and heat maps were used to describe the distribution of differentially expressed circRNAs (pval < 0.05) between patients with different prognoses.

### Quantitative reverse transcription-polymerase chain reaction (qRT-PCR) assay

Total RNA was extracted from GC tissues with TRIzol reagent (Invitrogen, Carlsbad, CA, USA) according to the manufacturer’s instructions. RNA integrity was verified by agarose gel electrophoresis, and the concentration and purity were measured with a NanoDrop spectrophotometer. A total of 500 ng of total RNA was reverse-transcribed with random primers using the PrimeScritTM RT reagent kit (Takara, Dalian, Liaoning, China). The obtained cDNA was analyzed by qRT-PCR with TB Green Premix Ex Taq II (Takara) on the QuantStudio 5 Real-Time PCR System (Thermo Fisher Scientific). The PCR conditions were as follows: 95 °C for 30 s, 40 cycles at 95 °C for 5 s and 62 °C for 34 s. Melting curves were generated at the end of amplification. β-actin was used as an internal reference gene, and the relative expression level of circRNAs was calculated by the − ΔCT method. Divergent primers for circRNAs were designed for their back-splicing sites and synthesized by Sangon (Shanghai, China). Primer sequences were as follows: hsa_circ_0005092 forward 5′-GGCCAGATGAAGAAGGTAGTGAT-3′, hsa_circ_0005092 reverse 5′‐ACAGGTCTGATGAATGGTGTG‐3′, hsa_circ_0002647 forward 5′‐TGACCTGAGACACCTATGGC‐3′, reverse 5′‐TAGTGTGTTGGTGCCATCCT‐3′. Other primers are shown in Additional file 4: Table S2. The accuracy of the amplified product was confirmed by agarose gel electrophoresis and Sanger sequencing.

### Actinomycin D treatment

GC cells (5 × 10^4^, SNU-1) were seeded in 24-well plates and cultured with 5% CO_2_ at 37 °C. After 24 h, actinomycin D was added to the wells at a final concentration of 2ug/mL for 0 h, 3 h, 6 h, 9 h, and 12 h. The cells at each time point were harvested and extracted for RNA. qRT-PCR was used to detect the relative expression of circRNAs and their host genes. Primer sequences were as follows: IPO7 (the host gene of hsa_circ_0005092) forward 5′-ATCGAGAAACAGCACCAGGG-3′, reverse 5′-CTACCAATGGACTCCGCTCC-3′; EHMT1 (the host gene of hsa_circ_0002647) forward 5′-GCTGTGTGAAAACCGAGCTG-3′, reverse 5′-TCCGCTATCCGAGTTAGTGTG‐3′.

### RNase R treatment

A total of 2 μg RNA from SNU-1 and MKN-45 cells was incubated with or without 6 U of RNase R (Epicentre Technologies, Madison, WI, USA) for 15 min at 37 °C and 10 min at 70 °C. The resulting RNA in equal volume were used for qRT-PCR to evaluate the stability of circRNAs. β-actin was used as an internal reference gene.

### Extraction and expression validation of circRNA in plasma

The plasma of 29 GC patients and 17 healthy people were collected in this experiment. 200 μl plasma per sample was used for RNA extraction by EZ-press RNA Purification Kit (EZBioscience, Roseville, US). The resulting RNA in equal volume were used for qRT-PCR to detect the expression level of hsa_circ_0005092 and hsa_circ_0002647. β-actin was used as an internal reference gene.

### CeRNA network analysis and function annotation

The potential interactions between circRNAs and miRNAs were predicted by the CircInteractome (https://circinteractome.nia.nih.gov/index.html) website. For each circRNA, 6 miRNAs with more binding sites and lower context scores were selected for further analysis. Target mRNAs of these miRNAs were predicted on the multimiR tooland intersected using Venn diagrams. Cytoscape was used to delineate the circRNA-miRNA-mRNA network. To further understand the potential functions of circRNAs, gene ontology (GO) and Kyoto Encyclopedia of Genes and Genomes (KEGG) analyses were performed on the ceRNA regulatory network of circRNAs.

### Statistical analysis

All of the statistical data were analyzed using SPSS software, version 21.0 (SPSS, Chicago, IL, USA), and GraphPad software, verson 7.0 (GraphPad Software, San Diego, CA, USA). Kaplan–Meier estimator was performed to evaluate the prognostic value of circRNAs for RFS and OS in GC patients. Cox regression analysis was used to screen the prognostic risk factors for GC and establish a risk assessment model for postoperative recurrence. Receiver operating characteristic (ROC) curves were generated, and the area under the ROC curve (AUC) was measured to reflect the accuracy of the model with the Youden index (specificity + sensitivity−1). The chi-square test was used to analyze the relationships between circRNA levels and clinicopathological factors in GC patients. *p* values < 0.05 were considered to be significant.

## Results

### circRNA profiles in GC patients with different prognoses

**Fig. 1:**
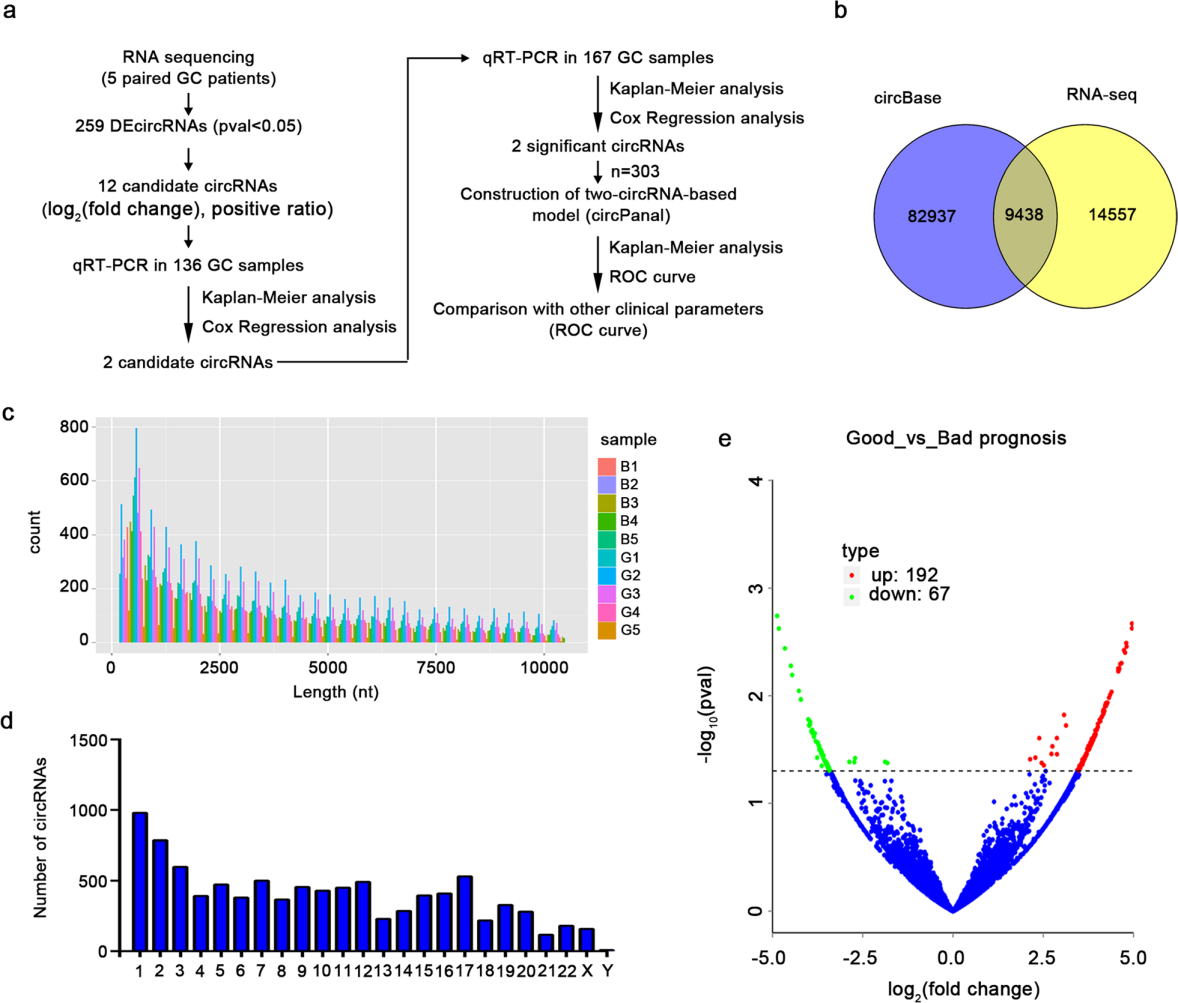
circRNA expression profile of GC patients with different prognoses. **a** The flowchart for circRNA screening in this study. **b** Annotation of circRNAs obtained by sequencing in circBase. **c** The length distribution of circRNAs. **d** Chromosomal distribution of circRNAs which have been annotated in circBase. **e** The expression profiling of differentially expressed circRNAs between GC patients with good and bad prognoses. Red points: upregulated circRNAs; green points: downregulated circRNAs; blue points: circRNAs with no statistical significance. GC, gastric cancer; circRNA, circular RNA; qRT-PCR, quantitative reverse transcription-polymerase chain reaction; ROC curve, receiver operating characteristic curve

The flowchart for circRNA screening in this study is shown in Fig. 1a. We performed RNA sequencing to characterize the circRNA expression profiles of GC patients with different RFS. A total of 23,995 circRNAs were detected in the discovery set, among which 9443 circRNAs were included in the circBase database and annotated (Fig. 1b). Consistent with previous studies, these circRNAs were distributed in all human chromosomes, and their splicing length was mostly less than 1000 nucleotides (Fig. 1c, d). Differential analysis was performed on the circRNA expression profile of GC patients with different prognoses. A total of 259 differentially expressed circRNAs were identified with the threshold of pval < 0.05, of which 192 circRNAs were upregulated, and 67 circRNAs were downregulated in GC patients with good prognoses (Fig. 1e).

### Screening and validation of prognostic circRNAs

**Fig. 2:**
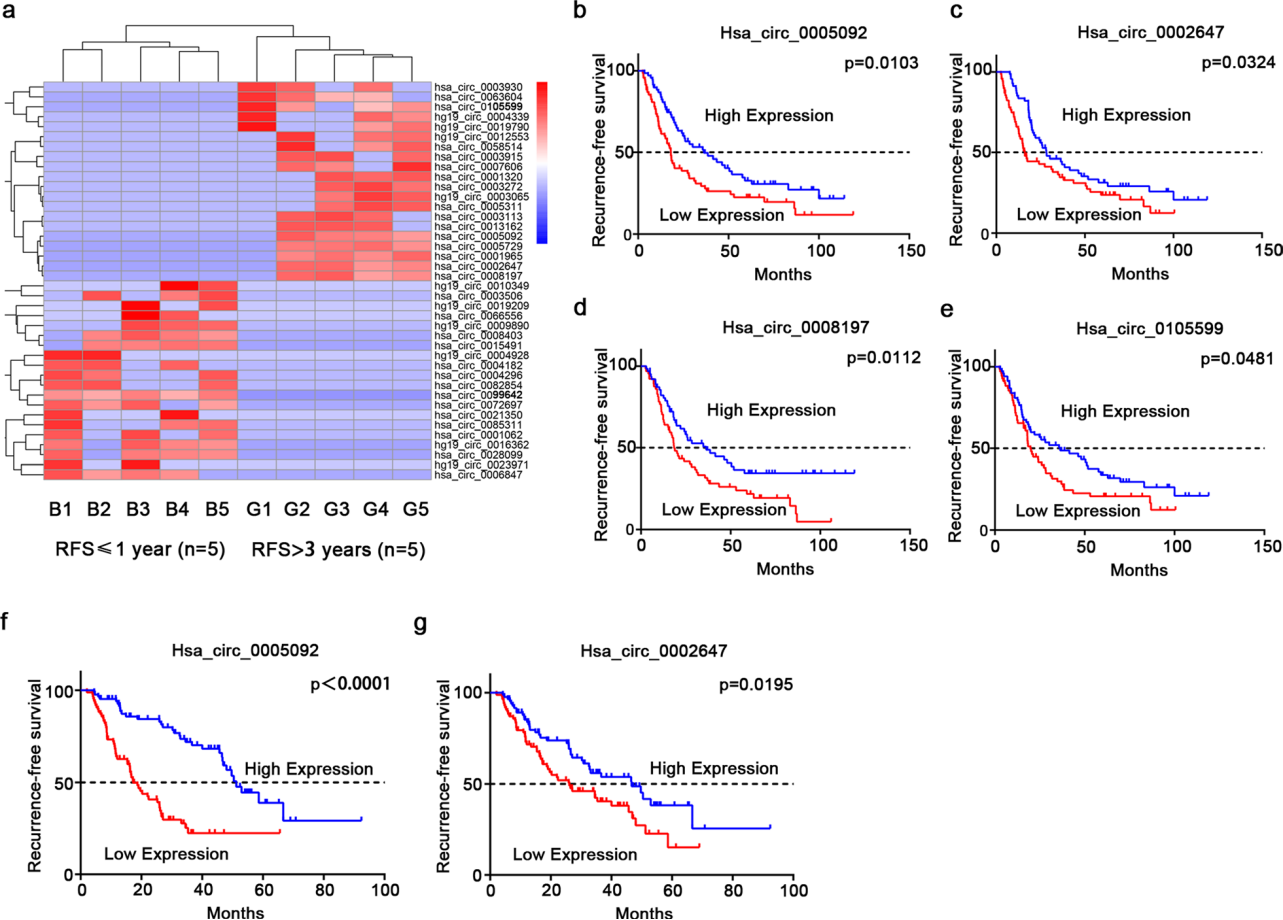
Selection and validation of candidate circRNAs. **a** Heat map of the top 20 up- and downregulated circRNAs. G1-G5: GC patients with good prognoses; B1-B5: GC patients with bad prognoses; red strip: high relative expression; blue strip: low relative expression. **b**-**e** Kaplan–Meier survival analysis of hsa_circ_0005092, hsa_circ_0008197, hsa_circ_0002647 and hsa_circ_0105599 in the training set (n = 136). Blue line: high expression; red line: low expression. **f**, **g** Kaplan–Meier survival analysis of hsa_circ_0005092, hsa_circ_0002647 in the validation set (n = 167). Blue line: high expression; red line: low expression. RFS, recurrence-free survival

Then, we evaluated whether circRNAs could be used as prognostic biomarkers in GC patients. The top 20 up- and downregulated circRNAs with extremely fold change obtained by RNA sequencing were included in the preliminary screening (Fig. 2a). Considering the contingency of RNA sequencing samples, 12 circRNAs with significant expression differences in at least 4 of 5 pairs of samples were selected for further validation. The circRNA ID, genome position, spliced length, and gene symbols of these 12 candidate circRNAs are shown in Table 1.

**Table 1:** The information of 12 candidate circRNAs

circRNA ID	Genome position	Strand	Spliced length	Gene name	Log2FC	P Value	Regulation
Hsa_circ_0005729	chr18:29691716–29693823	+	282	RNF138	5.974	9.58E−05	Up
Hsa_circ_0005092	chr11:9451220–9452550	+	290	IPO7	4.9603	0.002134	Up
Hsa_circ_0001965	chr3:169854206–169863309	–	346	PHC3	4.7984	0.003229	Up
Hsa_circ_0002647	chr9:140611077–140646860	+	1163	EHMT1	4.7371	0.003767	Up
Hsa_circ_0063604	chr22:42204878–42206004	+	241	CCDC134	4.6347	0.00505	Up
Hsa_circ_0105599	chr16:53967896–53971745	+	3849	FTO	4.5821	0.005785	Up
Hsa_circ_0008197	chr1:51032748–51061888	–	524	FAF1	4.5784	0.005578	Up
Hsa_circ_0099642	chr12:9833518–9847565	+	692	CLEC2D	− 4.8723	0.001811	Down
Hsa_circ_0006847	chr16:29916172–29917280	+	286	ASPHD1	− 4.8243	0.002375	Down
Hsa_circ_0072697	chr5:64863339–64868113	+	773	PPWD1	− 4.6578	0.003623	Down
Hsa_circ_0015491	chr1:180047597–180049796	+	357	CEP350	− 4.4914	0.005258	Down
Hsa_circ_0008403	chr5:141732790–141733148	+	358	TCONS_l2_00023557	− 4.268	0.008983	Down

*FC* fold change

The relative expression levels of 12 circRNAs were detected by qRT-PCR assay in the training set (n = 136). According to the median expression level of each circRNA, GC patients were divided into high and low expression groups. Kaplan–Meier survival analysis showed that four circRNAs (hsa_circ_0005092, hsa_circ_0002647, hsa_circ_0008197 and hsa_circ_0105599) were significantly associated with RFS in GC patients and were upregulated in patients with good prognoses, which was consistent with the sequencing results (Fig. 2b–e, Additional file 1: Figure S1). Among them, hsa_circ_0005092 and hsa_circ_0002647 were confirmed to be independent prognostic factors of RFS in GC patients by univariate and multivariate Cox regression analysis (Table 2).

**Table 2:** Univariate and multivariate Cox regression analysis of prognostic factors for RFS in the training set

Factors	Univariate analysis		Multivariate analysis	
	HR (95% CI)	P value	HR (95% CI)	P value
Age	0.997 (0.976–1.018)	0.774	1.010 (0.987–1.034)	0.405
Sex	1.221 (0.730–2.043)	0.446	1.095 (0.637–1.885)	0.742
Tumor size	1.024 (0.940–1.114)	0.589	0.998 (0.901–1.105)	0.967
Hsa_circ_0005092	1.531 (1.219–1.922)	0.000***	1.784 (1.256–2.534)	0.001**
Hsa_circ_0008197	1.121 (0.942–1.334)	0.198	0.960 (0.811–1.137)	0.637
Hsa_circ_0002647	1.345 (1.139–1.589)	0.001**	1.294 (1.072–1.563)	0.007**
Hsa_circ_0105599	1.176 (1.003–1.378)	0.045*	0.872 (0.706–1.078)	0.206

*RFS* recurrence-free survival, *HR* Hazard ratio; *CI* confidence interval

*p < 0.05, **p < 0.01, ***p < 0.001.

**Fig. 3:**
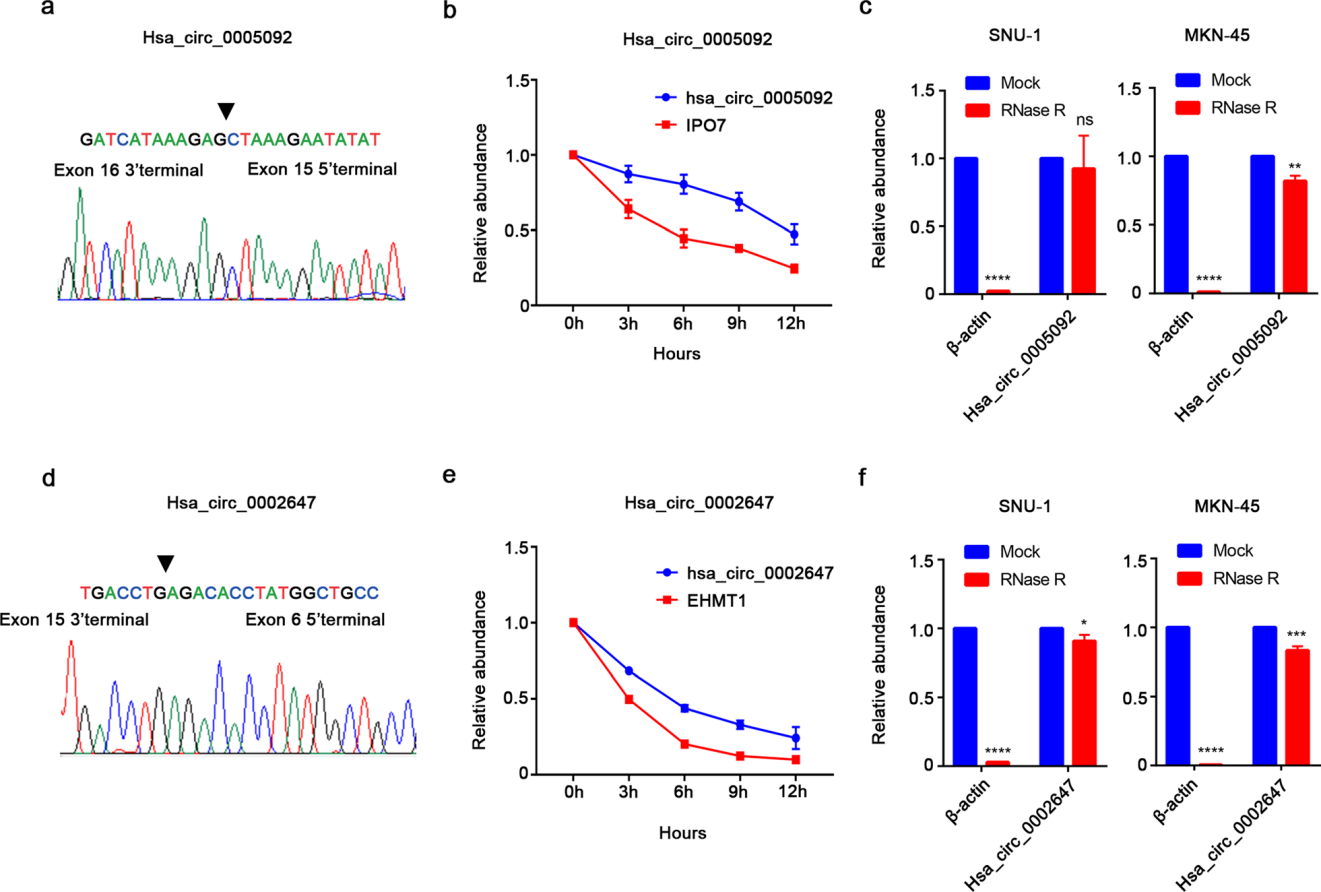
Characterization of hsa_circ_0005092 and hsa_circ_0002647. **a** The RT-PCR product of the hsa_circ_0005092 is verified by sanger sequencing. **b** qRT–PCR analysis for the expression of hsa_circ_0005092 and its host gene after actinomycin D treatment for 0 h, 3 h, 6 h, 9 h, and 12 h in SNU-1 cells. **c** RNase R resistance assay for has_circ_0005092 in SNU-1 and MKN-45 cells. **d** The RT-PCR product of the hsa_circ_0002647 is verified by sanger sequencing. **e** qRT–PCR analysis for the expression of hsa_circ_0002647 and its host gene after actinomycin D treatment. **f** RNase R resistance assay for has_circ_0002647 in SNU-1 and MKN-45 cells. IPO7, importin 7; EHMT1, euchromatic histone lysine methyltransferase 1

To further verify the correlations of hsa_circ_0005092 and hsa_circ_0002647 with postoperative recurrence in GC patients, a validation set (n = 167) was applied. Survival analysis showed that GC patients with high expression level of hsa_circ_0005092 and hsa_circ_0002647 had longer RFS than those with low expression (Fig. 2f-g). Regression analysis also confirmed the prognostic value of hsa_circ_0005092 and hsa_circ_0002647 (Table 3). Additionally, the circularity of the two circRNAs was verified by Sanger sequencing (Fig. 3a, d), and their half-lives were experimentally demonstrated to be longer than those of linear host genes (Fig. 3b, e). RNase R resistance assays also confirmed that they both have circular structure and higher stability (Fig. 3c, f). More importantly, we also detected their presence in the plasma of GC patients and healthy people (Additional file 2: Figure S2). Taken together, the above results indicated that hsa_circ_0005092 and hsa_circ_0002647 have the potential to be biomarkers for postoperative recurrence in GC patients.

**Table 3:** Univariate and multivariate Cox regression analysis of prognostic factors for RFS in the validation set

Factors	Univariate analysis		Multivariate analysis	
	HR (95% CI)	P value	HR (95% CI)	P value
Age	1.003 (0.980–1.027)	0.782	1.003 (0.979–1.028)	0.826
Sex	0.948 (0.604–1.486)	0.914	0.935 (0.577–1.516)	0.786
Tumor size	0.967 (0.894–1.046)	0.405	0.996 (0.919–1.079)	0.916
Hsa_circ_0005092	1.831 (1.388–2.416)	0.000***	1.437 (1.015–2.033)	0.041*
Hsa_circ_0002647	1.979 (1.475–2.655)	0.007**	1.565 (1.066–2.300)	0.022*

*RFS* recurrence-free survival, *HR* Hazard ratio, *CI* confidence interval

*p < 0.05, **p < 0.01, ***p < 0.001

**Table 4:** Correlation between circRNAs and clinicopathologic features of GC patients

Variables	Group	Cases	circPanel		
			High expression	Low expression	P value
		n = 303	n = 151	n** = **152	
Age	≤ 60 years	155	73	88	0.0958
	> 60 years	148	78	64	
Sex	Male	222	111	111	0.9242
	Female	81	40	41	
Tumor size	≤ 5 cm	143	80	73	0.3885
	> 5 cm	150	71	79	
T stage	T1-3	41	26	15	0.0359*
	T4a	181	93	88	
	T4b	82	32	49	
N stage	N0	73	36	37	0.1369
	N1	73	41	32	
	N2	76	42	34	
	N3	81	32	49	
TNM stage	I–II	75	40	35	0.1041
	IIIA	113	64	49	
	IIIB	63	26	38	
	IIIC	52	21	30	
Differentiation	Poor-undifferentation	158	69	89	0.0251*
	Well-moderate differentation	145	82	63	

*p < 0.05

### Construction of prognostic model based on hsa_circ_0005092 and hsa_circ_0002647

Next, the training set and validation set were combined to construct a postoperative recurrence model based on the regression coefficients of hsa_circ_0005092 and hsa_circ_0002647 for statistical significance (Additional file 4: Table S3):

$${\text{circPanel}} = - 0.369 \times Exp_{hsa\_circ\_0005092} - 0.223 \times Exp_{hsa\_circ\_0002647}$$

**Fig. 4:**
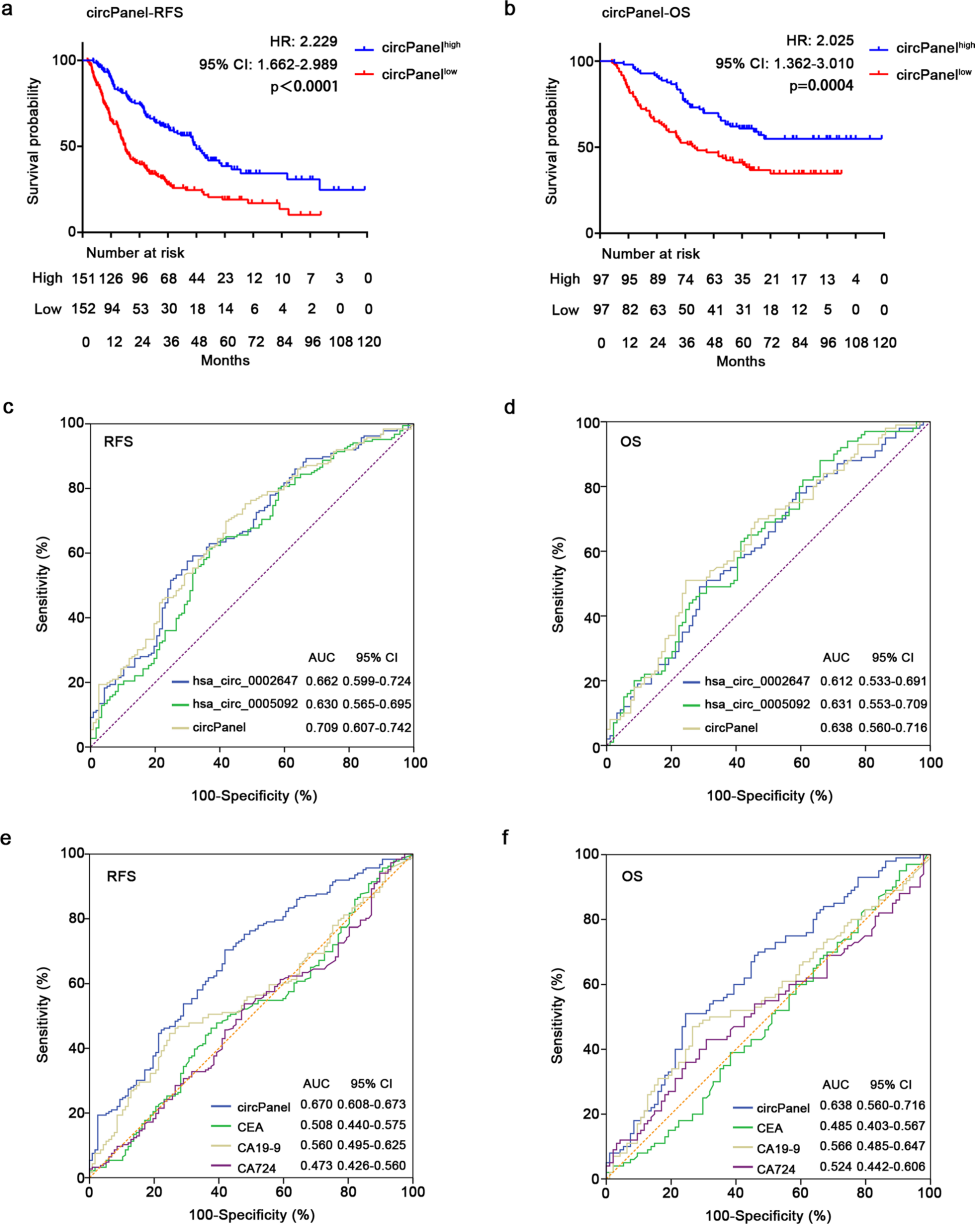
Construction and validation of circPanel. **a** Kaplan–Meier survival analysis of circPanel on RFS of GC patients, n = 303. **b** Kaplan–Meier survival analysis of circPanel on OS of GC patients, n = 194. **c** ROC curves analysis of hsa_circ_0002647, hsa_circ_0005092 and circPanel on RFS in GC patients. **d** ROC curves analysis of hsa_circ_0002647, hsa_circ_0005092 and circPanel on OS in GC patients. RFS, recurrence-free survival; OS, overall survival; HR, hazard ratio; CI, confidence interval; ROC, receiver operator characteristic; AUC, area under the curve

The expression level of hsa_circ_0005092 and hsa_circ_0002647 was brought into the circPanel for calculation to obtain the recurrence risk index of each patient. A total of 303 gastric cancer patients were included in the statistics, and were divided into high- and low-risk groups according to the median of the recurrence risk index obtained above. Patients with a high recurrence risk index were defined as circPanel^high^ patients, and patients with a low recurrence risk index were defined as circPanel^low^ patients. Survival analysis showed that circPanel^low^ patients had a shorter RFS than circPanel^high^ patients (hazard ratio [HR]: 2.229, 95% confidence interval [CI]: 1.662–2.989, *p* < 0.0001, Fig. 4a). The ROC curve and the area under the ROC curve (AUC) were further used to analyze the prognostic value of circPanel, as shown in Fig. 4c. Compared with the hsa_circ_0005092 and hsa_circ_0002647, circPanel had a larger AUC (0.709, 95% CI 0.607–0.742), with sensitivity of 69.9% and specificity of 58.1%.

Moreover, we observed a similar effect of circPanel on OS in GC patients. Patients with circPanel^low^ had a shorter OS (HR: 2.025, 95% CI 1.362–3.010, *p* = 0.0004, Fig. 4b). As shown in Fig. 4d, the AUCs of hsa_circ_0002647, hsa_circ_0005092 and circPanel were 0.612 (95% CI 0.533–0.691), 0.631 (95% CI 0.553–0.709) and 0.638 (95% CI 0.56–0.716), respectively. The sensitivity and specificity of circPanel for predicting OS were 51.0% and 75.5%, respectively. In short, circPanel could be used as a biomarker for the prognostic evaluation of GC patients, with better predictive performance than single circRNA.

### Comparative analysis of circRNA and other clinical indicators

We also analyzed the correlation between circPanel and the clinicopathological features of GC patients. The results suggested that circPanel was related to the tumor differentiation and T stage in GC patients (Table 4). Patients with circPanel^high^ might have higher tumor differentiation and lower T stages. No significant association was observed between circPanel and other characteristics (including sex, age, tumor size, N stage and TNM stage).

In addition, we compared the prognostic value of circPanel with some traditional tumor markers (including CEA, CA19-9 and CA724) by ROC curve analysis. As a result, the AUCs of circPanel, CEA, CA19-9 and CA724 were 0.67, 0.508, 0.56, and 0.493 for RFS, respectively, and 0.638, 0.485, 0.566, and 0.524 for OS. CircPanel has greater prognostic value than CEA, CA19-9 and CA724 (Fig. 4e, f). In summary, these results strongly confirmed the clinical application value of circPanel in the prognostic assessment of GC patients.

### CeRNA network of hsa_circ_0005092 and hsa_circ_0002647

**Fig. 5:**
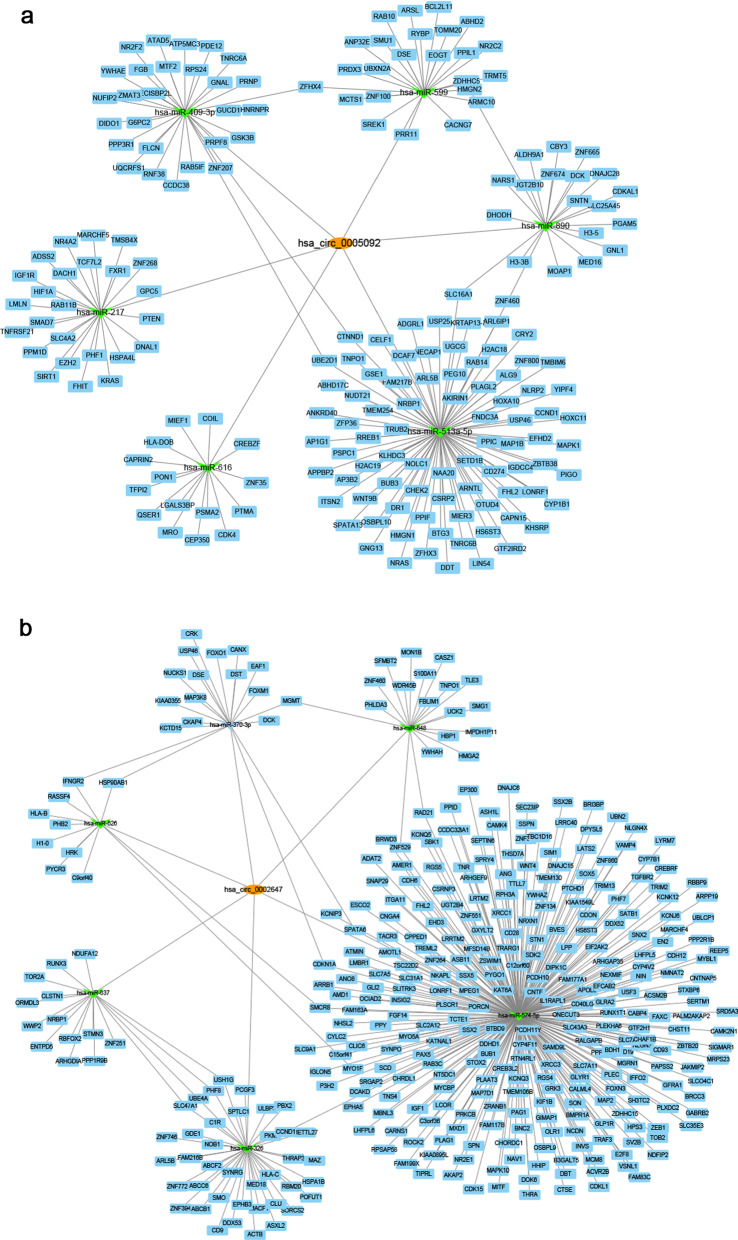
CeRNA network of hsa_circ_0005092 and hsa_circ_0002647. The circRNA-miRNA-mRNA networks of **a** hsa_circ_0005092 and **b** hsa_circ_0002647. The networks were predicted using CircInteractome and multimiR, and visualized using Cytoscape software. The orange ellipse nodes represent circRNAs, while the green arrow nodes represent miRNAs and the blue rectangular nodes represent mRNAs

Furthermore, we attempted to explore the biological function of these two selected circRNAs. At present, ceRNA is the most common circRNA regulation mechanism. Through the prediction of the CircInteractome, 6 miRNAs (miR-616, miR-599, miR-409-3p, miR-217, miR-513a-5p and miR-890) targeted by hsa_circ_0005092 and 6 miRNAs (miR-370, miR-626, miR-637, miR-648, miR-326 and miR-574-5p) targeted by hsa_circ_0002647, with more binding sites and lower context scores, were screened. Then, we predicted the target mRNAs of these miRNAs by multimiR and obtained 193 and 372 mRNAs for hsa_circ_0005092 and hsa_circ_0002647 (Additional file 4: Table S4), respectively. Cytoscape was further used to describe the regulatory network of circRNA-miRNA-mRNA (Fig. 5a, b).

### GO and KEGG analysis of hsa_circ_0005092 and hsa_circ_0002647

**Fig. 6:**
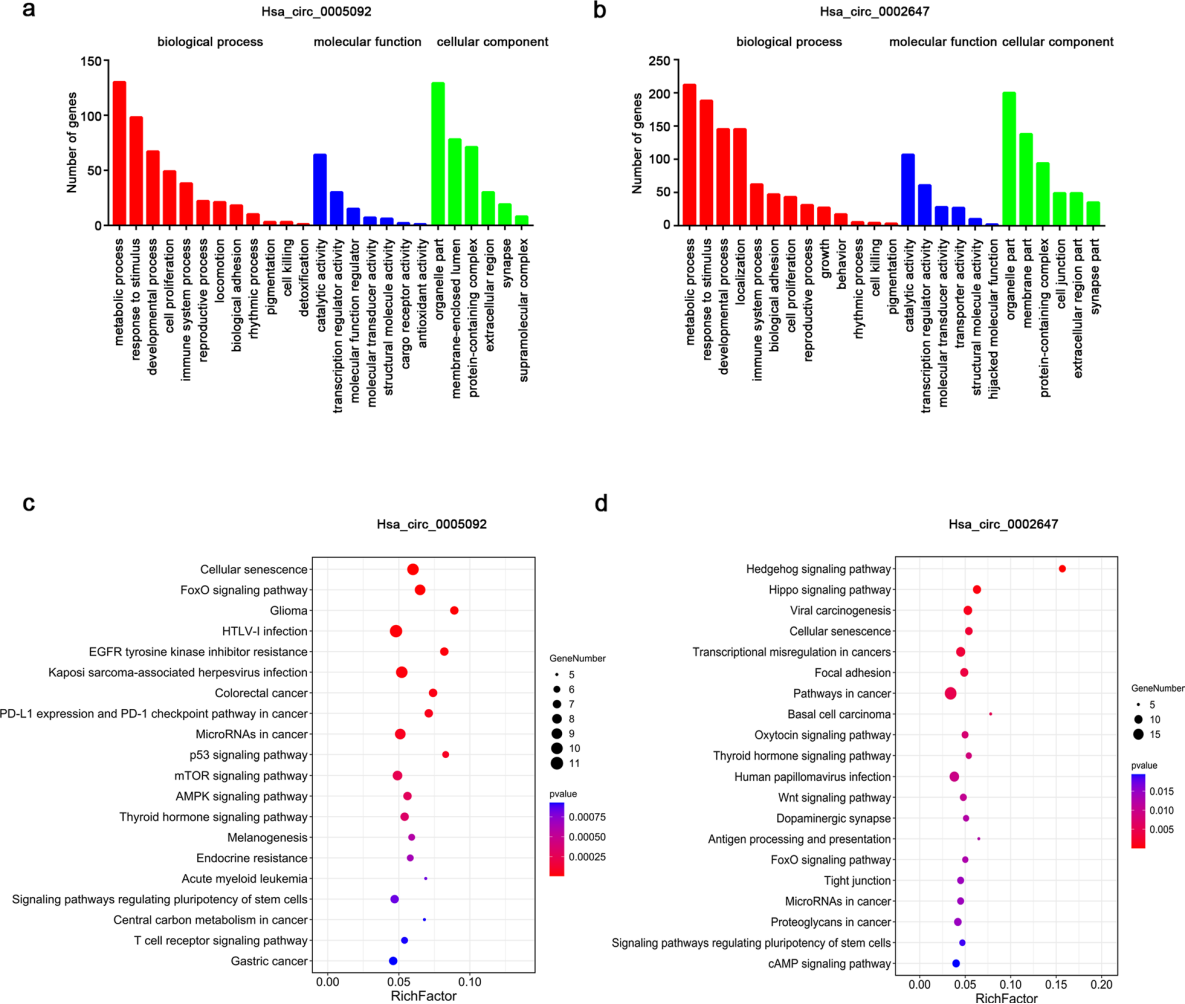
Function annotations of hsa_circ_0005092 and hsa_circ_0002647. The biological process (red), molecular function (blue) and cellular component (green) of hsa_circ_0005092 (**a**) and hsa_circ_0002647 (**b**) were predicted by GO enrichment analysis with the criteria of pvalue < 0.05. The signaling pathways of hsa_circ_0005092 (**c**) and hsa_circ_0002647 (**d**) were assessed by KEGG enrichment analysis. The size of the dot presents the count of genes; the gradation of color presents -log10 (FDR)

To further predict the potential function of circRNAs, GO and KEGG enrichment analyses were performed on the ceRNA regulatory networks of these two circRNAs. The significantly enriched biological processes (BPs), cellular components (CCs) and molecular functions (MFs) are shown in Fig. 6a, b. The main biological function of the hsa_circ_0005092 and hsa_circ_0002647 both included the involvement of metabolic process, response to stimulus and developmental process. In addition, hsa_circ_0005092 may also be involved in cell proliferation, while hsa_circ_0002647 involved in molecular localization and cell adhesion. The pathways enriched for the two circRNAs based on KEGG analysis are shown in Fig. 6c, d. The cellular senescence, FoxO signaling pathway, and microRNAs in cancer involved in hsa_circ_0005092 and the Hippo signaling pathway, cellular senescence and transcriptional misregulation in cancers involved in hsa_circ_0002647 are all related to cancer.

## Discussion

Although screening methods and treatment strategies have made great progress in recent years, the mortality rate of GC patients remains high. Radical surgical resection remains the only possible curative treatment option for GC patients. However, unfortunately, postoperative recurrence of GC patients is very common. Generally, recurrence within 2 years after surgery is defined as early recurrence, and after more than 2 years, it is defined as late recurrence. In China, approximately 42.5% of GC patients experience early recurrence [15], indicating a poor prognosis. Postoperative recurrence assessment plays an important role in guiding clinical medication and improving prognosis in gastric cancer [16, 17]. Currently, tumor size, invasion depth, pathological type, number of metastatic lymph nodes and some tumor markers (CEA, CA19-9 and CA72-4) are used as predictors of GC recurrence clinically. However, it is difficult to accurately predict early recurrence by relying solely on these indicators.

The development of molecular biology has provided more possibilities for disease assessment. circRNAs have become potential biomarkers due to their high stability, specificity and conservation [18]. Researches on circRNAs as prognostic markers for GC patients are gradually being performed. For example, Tang et al. found that the expression of circ-KIAA1244 in plasma was negatively correlated with TNM staging and lymph node metastasis, and GC patients with low circ-KIAA1244 expression often had shorter overall survival [19]. Chen et al. also considered circPVT1 to be a novel proliferative factor and prognostic biomarker in GC [20].

In our study, we identified the differential expression profiles of circRNAs in GC patients with different prognoses by RNA high-throughput sequencing, and confirmed that hsa_circ_0005092 and hsa_circ_0002647, which were downregulated in patients with shorter RFS, are independent prognostic factors in GC patients. Based on these two circRNAs, we constructed a postoperative recurrence model, named circPanel. CircPanel was correlated with the degree of tumor differentiation and T stage, which are important prognostic factors for GC. Via validation in 303 samples, circPanel was proved to be able to distinguish GC patients with different prognoses with better predictive accuracy than some tumor markers (CEA, CA19-9 and CA72-4). Patients with circPanel^low^ had shorter RFS and OS. Therefore, circPanel is an effective and promising biomarker for the assessment of postoperative recurrence and the prognoses of GC patients. Timely and effective intervention in patients at risk of early recurrence predicted by circPanel is expected to greatly improve the prognoses of patients.

However, our study also has some limitations. First, due to the difficulty in obtaining tissue samples, we were unable to monitor the circRNA levels during treatment dynamically, preventing further study. Second, the sample size in our study could not meet the requirements of grouping patients according to their treatment strategies for statistics, making it impossible to eliminate interference from the treatment strategies of patients. Despite these limitations, the clinical significance of circPanel in the prediction of postoperative recurrence and the prognoses of gastric cancer cannot be denied. Further multicenter prospective studies with larger sample sizes are needed to verify the effectiveness of circPanel and find the optimal cutoff values for clinical use.

Furthermore, we also performed an in-depth study of the two screened circRNAs. Hsa_circ_0005092 and hsa_circ_0002647 were derived from the back-splicing of importin 7 (IPO7) and euchromatic histone lysine methyltransferase 1 (EHMT1) respectively, with lengths of 290 bp and 1163 bp. Considering that miRNA sponges are among the main mechanisms for circRNAs [21],we predicted their ceRNA regulatory networks. The ceRNA network of hsa_circ_0005092 contains 6 miRNAs and 193 mRNAs, while the ceRNA network of hsa_circ_0002647 includes 6 miRNAs and 372 mRNAs. These two circRNAs could have a wide range of biological effects on the metabolic process, response to stimulus, developmental process, cell proliferation and adhesion. However, it should be noted that this ceRNA regulatory network was predicted by bioinformatics tools but not experimentally verified which is also the limitation of this study. Additionally, in our study, both hsa_circ_0005092 and hsa_circ_0002647 were highly expressed in GC patients with longer RFS and OS and were correlated with tumor differentiation and T stage, suggesting that these two circRNAs might be negatively associated with the malignant behavior of GC. This trend can be explained by previous studies. For example, multiple reports have shown that circRNAs tent to upregulate in the nervous system, which may be related to their post-transcriptional accumulation in neurons [22, 23]. And according to the sequencing data, the content of circRNA in tumor samples is often lower than that in normal samples and negatively correlated with the cell proliferation index [24]. Considering the relationship between drug resistance and tumor recurrence [25, 26], we speculated that these two circRNAs might be involved in the regulation of cell proliferation, cell invasion and drug resistance. However, the detailed functions and mechanisms of hsa_circ_0005092 and hsa_circ_0002647 must still be further studied.

## Conclusions

In conclusion, we described the circRNA expression profiles of GC and identified 2 upregulated circRNAs (hsa_circ_0005092 and hsa_circ_0002647) in patients with good prognoses. The circPanel, calculated based on these two circRNAs, was confirmed to be positively correlated with RFS and OS in GC patients. CircPanel could serve as a potential biomarker for prognostic evaluation in GC patients.

## Supplementary Information

**Additional file 1:Figure S1.** Selection of candidate circRNAs. Kaplan-Meier survival analysis of hsa_circ_0006847 (a), hsa_circ_0008403 (b), hsa_circ_0015491 (c), hsa_circ_0072697 (d), hsa_circ_0063604 (e), hsa_circ_0001965 (f), hsa_circ_0099642 (g) and hsa_circ_0005729 (h) in the training set (n=136). Blue line: high expression; red line: low expression.

**Additional file 2:Figure S2.** Expression of hsa_circ_0005092 and hsa_circ_0002647 in plasma of GC patients and healthy people. a GC patients(n=29) were divided into two groups according to RFS: RFS < 2 years(n=13) and RFS > 2 years(n=16). The expression of hsa_circ_0005092 in the plasma of GC patients was analyzed by qRT–PCR. b qRT–PCR analysis for the differentially expression of hsa_circ_0005092 in plasma of GC patients and healthy people(n=17). c qRT–PCR analysis for the expression of hsa_circ_0002647 in plasma of GC patients with different prognoses. d qRT–PCR analysis for the differentially expression of hsa_circ_0002647 in plasma of GC patients and healthy people.

**Additional file 3:Figure S3.** The error rate of each sample in RNA sequencing.

**Additional file 4:Table S1.** Clinical characteristic of gastric cancer patients involved in this study. **Table S2.** The divergent primers of 12 candidate circRNAs for qRT-PCR. **Table S3.** The primers of host genes of hsa_circ_0005092 and hsa_circ_0002647. **Table S4.** miRNAs with the potential to bind to has_circ_0005092 and hsa_circ_0002647.

## Data Availability

All data generated or analysed during this study are included in this published article.

## Abbreviations

circRNA: Circular RNA
RFS: Recurrence-free survival
OS: Overall survival
CEA: Carcinoembryonic antigen
CA19-9: Carbohydrate antigen 19-9
CA724: Carbohydrate antigen 724
miRNA: MicroRNA
TPM: Transcripts per million
qRT-PCR: Quantitative reverse transcription-polymerase chain reaction
ceRNA: Competing endogenous RNA
GO: Gene ontology
KEGG: Kyoto Encyclopedia of Genes and Genomes
ROC: Receiver operating characteristic
AUC: Area under the ROC curve
BP: Biological process
CC: Cellular component
MF: Molecular function
IPO7: Importin 7
EHMT1: Euchromatic histone lysine methyltransferase 1
