# Supplementary figures and images for: Development of a two-circular RNA panel as potential prognostic biomarker for gastric cancer
Source: J Transl Med. 2021 Oct 2;19:412. doi: 10.1186/s12967-021-03075-y (PMC8487552; doi:10.1186/s12967-021-03075-y)

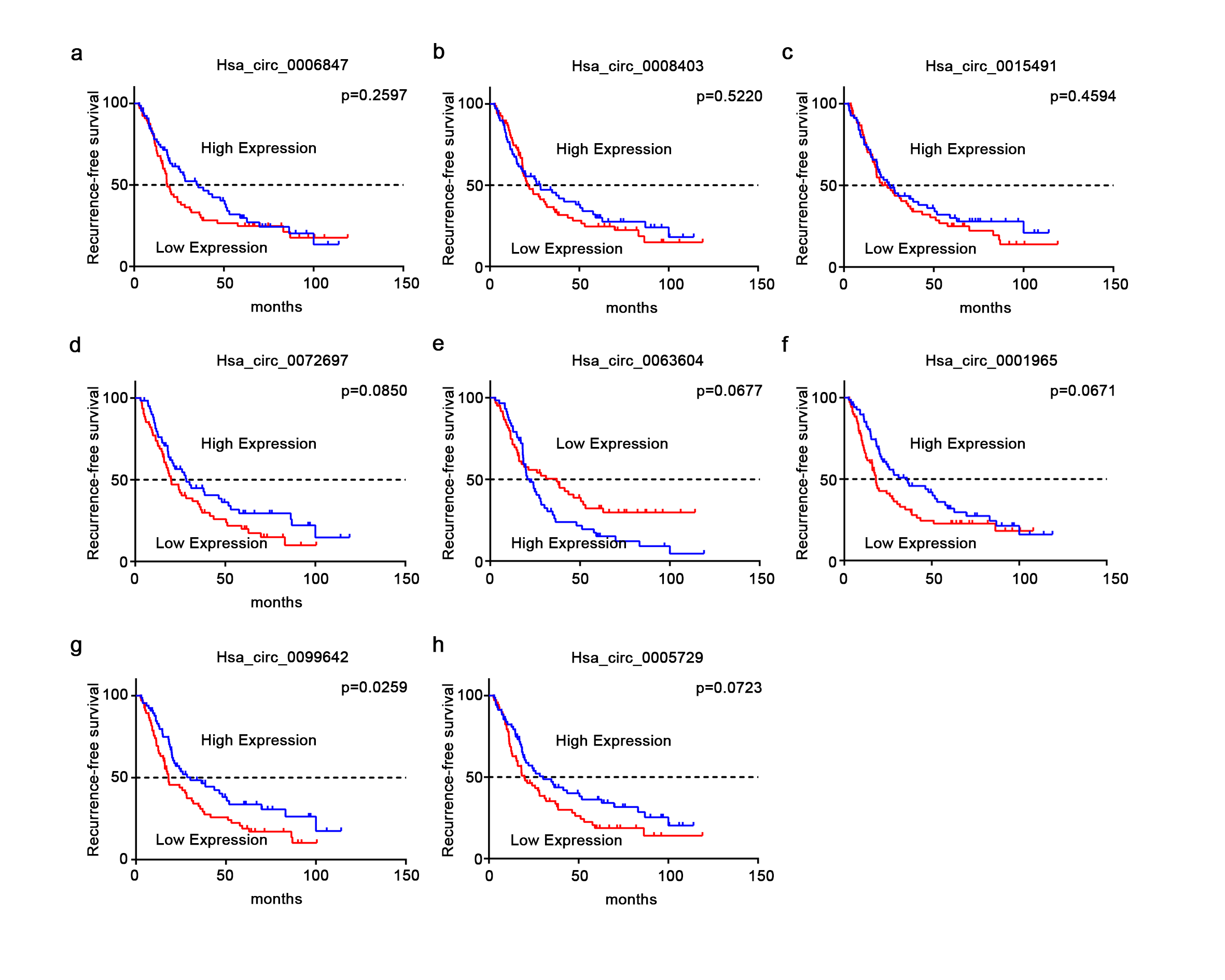

Supplement: Supplementary file 1 — Additional file 1:Figure S1. Selection of candidate circRNAs. Kaplan-Meier survival analysis of hsa_circ_0006847 (a), hsa_circ_0008403 (b), hsa_circ_0015491 (c), hsa_circ_0072697 (d), hsa_circ_0063604 (e), hsa_circ_0001965 (f), hsa_circ_0099642 (g) and hsa_circ_0005729 (h) in the training set (n=136). Blue line: high expression; red line: low expression. [file 12967_2021_3075_MOESM1_ESM.tif]
